# Analysis of Association of Occupational Physical Activity, Leisure-Time Physical Activity, and Sedentary Lifestyle with Hypertension according to the Adherence with Aerobic Activity in Women Using Korea National Health and Nutrition Examination Survey 2016-2017 Data

**DOI:** 10.1155/2020/8943492

**Published:** 2020-02-14

**Authors:** Mikyung Ryu, Sol Lee, Ho Gym, Weon-Chil Baek, Heejin Kimm

**Affiliations:** ^1^Department of Sports and Health Science, College of Human-Centered Convergence, Kyonggi University, Suwon, Republic of Korea; ^2^Institute on Aging, Ajou University Medical Center, Suwon, Republic of Korea; ^3^Department of bbko Research Center, bbko Big-Data R&D, Seoul, Republic of Korea; ^4^Department of Health Policy and Management, Korea University, Seoul, Republic of Korea; ^5^Department of Epidemiology and Health Promotion, Graduate School of Public Health, Yonsei University, Seoul, Republic of Korea; ^6^Department of Epidemiology and Health Promotion, Institute for Health Promotion, Graduate School of Public Health, Yonsei University, Seoul, Republic of Korea

## Abstract

**Purpose:**

We investigated the association between occupational physical activity, leisure-time physical activity, and sedentary lifestyle with hypertension by adherence with aerobic exercise in middle-aged and elderly women.

**Methods:**

A cross-sectional analysis was performed using Korea National Health and Nutrition Examination Survey (KNHANES), a nationally representative data between 2016 and 2017. A total of 4,241 women aged 40 years or older were included. Hypertension diagnosed by physician and exercise status was asked by questionnaires.

**Results:**

Mean age of the participants was 58.4 (±11.4, range: 40∼80 years). There were 1,681 (39.6%) women in the aerobic activity adherence group. In the logistic regression analysis with adjustment for confounding factors, frequency of occupational physical activity (OPA) level (OR 1.931; *p*=0.048, in ≤4 per week group), walking frequency (OR 0.436; *p*=0.048, in ≤4 per week group), walking frequency (OR 0.436; *p*=0.048, in ≤4 per week group), walking frequency (OR 0.436; *p*=0.048, in ≤4 per week group), walking frequency (OR 0.436;

**Conclusions:**

In the aerobic activity adherence group, further research is needed to identify the influence of occupational physical activity. In the aerobic activity nonadherence group, decreasing sitting hours and increasing endurance exercise may be helpful.

## 1. Introduction

Hypertension has been a well-known condition causing cardiovascular disease, stroke, and chronic kidney disease [1–3], leading to premature mortality and finally death if not detected and treated within the appropriate period [4]. Hypertension is also a worldwide important public health issue and epidemiological matter [5]. Moreover, it has been reported that health-related quality of life is lower in people with diagnosed hypertension than those without diagnosed hypertension [6]. Moreover, the estimated number of adults with hypertension has increased over the years from 594 million in 1975 to 1.13 billion in 2015 [7]. Physical activity is known to be associated with decreased incidence of hypertension. It has showed benefits on blood pressure not only in people with normal blood pressure but also in patients with prehypertension or hypertension [8].

Physical training, particularly aerobic exercises, is recommended as an antihypertensive solution leading to significant blood pressure, body weight, body fat, and waist circumference reduction, along with insulin sensitivity and HDL cholesterol level improvement [9, 10]. Also, regular aerobic training has been reported to reduce blood pressure in both younger and older people with hypertension by decreasing aortic stiffness and enhanced flow-mediated arterial dilation mediated by increased nitric oxide released from endothelial cells lining these blood vessels, similar to blood pressure reductions by a single antihypertensive drug [11]. In previous studies, regular physical training such as walking, jogging, or swimming for 30 to 45 minutes can reduce blood pressure and is recommended by current American and European hypertension guidelines [12, 13]. These were leisure-time physical activity, which consistently showed a positive effect on cardiovascular outcomes, including hypertension [14, 15]. The negative consequences of sedentary lifestyles are also well established [16].

However, despite the obvious reduction of cardiovascular mortality in other reports [15], there has been controversy among studies on the effects of occupational physical activity due to the risk of anaerobic components or injuries, depending on the nature of the work [14, 17]. Few studies explored the association of occupational, leisure-time, and sedentary activities with hypertension diagnosed by physician in middle-aged and elderly women. It is unclear whether this association depends on the adherence with aerobic activity.

The aim of this study is to examine the association of occupational physical activity, leisure-time physical activity, and sedentary lifestyle with hypertension according to the adherence with aerobic exercise in a nationally representative sample of women over 40 years of age, using recent Korea National Health and Nutrition Examination Survey (KNHANES) Data.

## 2. Methods

### 2.1. Participants

In this study, a cross-sectional analysis was performed using the Korea National Health and Nutrition Examination Survey (KNHANES) data collected by the Korea Centers for Disease Control and Prevention (KCDC) from 2016 to 2017. KNHANES is a surveillance system to assess the health and nutritional status of population in the Republic of Korea.

KNHANES includes a health interview, health examination, and nutrition survey. The interview and examination are performed by trained medical staff and interviewers. A multistage stratified cluster sampling was done for the household unit selection. The Institutional Review Board of the KCDC approved the data collection with written informed consent forms from all participants. The details of the survey design and data resource were described on a profiles paper in 2014 [18]. In this study, women participants older than 40 years of age who completed both answers on hypertension diagnosis (yes or no) and aerobic activities (yes or no) were included (*n* = 4,241).

### 2.2. Variables

Hypertension was defined as “participants' answer on hypertension diagnosed by medical doctor” from health survey questionnaires. Participants with no response were excluded from the analysis. If the participants answered “yes” to the questions for moderate intensity physical activity (at least 2.5 hours per week) or “yes” for vigorous intensity physical activity (at least 1.5 hours per week), they were classified as “aerobic activity adherence group.” Those who failed to be classified as the adherence group were categorized as “nonadherence group.” If the participants answered “yes” to the questions for muscle strengthening exercises (MSE) such as push-up, sit-up, dumbbell, or other types of muscle exercises more than 2 days for the past week, they were classified as “adherence group.”

Sociodemographic characteristics including age, quartiles of equivalent income, and education (less than elementary school, less than middle school, less than high school, and more than university graduation) were included in the questionnaires. Smoking status was classified as never smoker, former smoker, or current smoker, and alcohol consumption was divided into 4 categories (never, no drink within recent 1 year, less than 4 times drink per a month, or more than 5 times per month). Sleeping hours were classified as 7 hours of sleep per day in weekdays as well as in weekends, respectively. For physical activities, each of vigorous or moderate intensity physical activity group was divided into two aspects, work (occupational physical activity, OPA) and leisure (leisure-time physical activity, LPA), with 3 subcategories (never, ≤4 days per week, or 5∼7 days per week), respectively. Sitting hours were classified as ≤6, 7∼12, or ≥13 hours per a day. Frequency of walking per week was divided into 3 categories (never, ≤4 days per week, or 5∼7 days per week).

### 2.3. Statistical Analysis

The data were expressed by numbers and percentages for describing the general characteristics of the study participants. The frequency in the adherence group and the nonadherence group was compared by the chi-square test. To determine the difference for clinical variables in each group, independent two-sample *t*-test was done. Odds ratios (ORs) and 95% confidence intervals (CIs) using binomial logistic regression were obtained to analyze an association between physical activity and hypertension in women with adjustment for other covariates. A *p* value less than 0.05 was used for statistical significance. IBM SPSS statistics 22.0 software (IBM Corp, Armonk, NY) was used.

## 3. Results

### 3.1. General Characteristics of Study Population

The mean age of the 4,241 women participants from KNHANES 2016-2017 data was 58.4 (±11.4, range: 40–80 years old). Among them, 29.1% (1,234) of participants had hypertension and 92.7% were never smokers. There were 1,681 (39.6%) women in the aerobic activity adherence group and 2,560 (60.4%) participants were in the nonadherence group. For muscle strengthening exercises adherence, 3,560 (83.9%) participants never did the exercise, 465 (11.0%) did 1–4 day per week, and 215 (5.1%) did more than 5 days per week. Most participants reported no vigorous intensity physical activity for both occupation (99.5%) and leisure time (94.6%). There was a similar trend for moderate intensity physical activity (Table 1).

### 3.2. General Characteristics and Physical Activity Status due to Aerobic Activity Adherence

Aerobic activity adherence showed a significant association with age groups (40–49, 50–59, 60–69, and more than 70 years) (Table 2). Quartiles of equivalent income and education were associated with aerobic activity adherence (*p* < 0.05), and hypertension also showed a significant association with aerobic activity adherence. OPA and LPA days per week were significantly associated with aerobic activity adherence (*p* < 0.05). There was a significant association between aerobic activity adherence and physical activity days with moving location. Sitting hours were significantly associated with aerobic activity adherence (*p* < 0.05). Sleeping hours per week were associated with aerobic activity adherence (*p* < 0.05). However, no association was found between sleeping hours during weekend and aerobic activity adherence. Alcohol consumption showed significant association aerobic activity adherence (*p* < 0.05), but smoking status showed no association.

### 3.3. Comparison of Clinical Characteristics according to the Aerobic Activity Adherence

Systolic blood pressure (SBP) and diastolic blood pressure (DBP) showed significant differences between the aerobic activity nonadherence group and aerobic activity adherence group (*p* < 0.05). Body mass index (BMI) and HDL cholesterol showed significant differences between the two groups (*p* < 0.05). In triglyceride, glutamate oxaloacetate transaminase (GOT), and glutamate-pyruvate transaminase (GPT) levels, there were significant differences between the two groups (*p* < 0.05). However, total cholesterol showed no difference (Table 3).

### 3.4. Association between Physical Activity and Hypertension in the Aerobic Activity Nonadherence Group

In the aerobic activity nonadherence group, the odds ratio (OR) for hypertension in muscle strengthening exercises (MSE) adherence group (1∼4 days per week) was 0.468 (95% CI: 0.322–0.681, *p* < 0.001) in the unadjusted model and 0.554 (95% CI: 0.353–0.870) in the adjusted model with adjustment for age, quartiles of equivalent income, education levels, smoking status, and alcohol consumption, compared with the “never” group.

For sitting hours, ORs for hypertension were 1.211 (95% CI: 1.008–1.455) in 7∼12 hours group and 2.275 (95% CI: 1.704–3.036) in 13 hours or longer group, compared with 6 hours or less sitting hours group in unadjusted model, respectively. They were 1.161 (95% CI: 0.929–1.451) and 1.849 (95% CI: 1.279–2.673) compared with 6 hours or less sitting hours group in the adjusted model with adjustment of age, quartiles of equivalent income, education levels, smoking status, and alcohol consumption, respectively.

A significant association was found between hypertension and days of walking per week. The OR for hypertension was 0.802 (95% CI: 0.644–0.998) in ≤4 days per week group, compared with the never walking group in the unadjusted model. However, no significant association was found with hypertension after adjustment.

Sleeping hours during week or weekend showed significant association with hypertension in the unadjusted model. However, the association disappeared in results from the adjusted model (Table 4).

### 3.5. Association between Physical Activity and Hypertension in the Aerobic Activity Adherence Group

In the aerobic activity adherence group who answered “yes” to the question about aerobic activity adherence, there was no significant association between MSE adherence and hypertension in both unadjusted model and adjusted model with adjustment forage, quartiles of equivalent income, education levels, smoking status, and alcohol consumption.

Frequency of leisure-time physical activity (LPA) level of vigorous and moderate intensity was associated with hypertension in the unadjusted model. The OR for hypertension was 0.537 (*p*=0.030) in ≤4 per week group and 0.314 (*p*=0.017) in 5∼7 per week group of vigorous intensity, respectively. They were 0.701 (*p*=0.032) in ≤4 per week group of moderate intensity, but it was not significant in 5∼7 per week group. The association was not significant in adjusted models.

Frequency of occupational physical activity (OPA) level of moderate intensity showed increased association with hypertension in the adjusted model (in ≤4 per week group, OR = 1.931; *p*=0.048).

Days of walking per week were significantly associated with hypertension. The OR for hypertension was 0.410 (95% CI: 0.243–0.692, *p*=0.001) in ≤4 per week group and 0.436 (95% CI: 0.265–0.718) in 5∼7 days per week group compared with the never walking group in the unadjusted model, respectively.

They were 0.532 (95% CI: 0.284–0.997) and 0.530 (95% CI: 0.292–0.963) compared with the never walking group in the adjusted model with adjustment of age, quartiles of equivalent income, education levels, smoking status, and alcohol consumption, respectively.

However, sitting hours per day and sleeping hours during week showed no significant association with hypertension in each model (Table 5).

## 4. Discussion

In the aerobic activity nonadherence group, women who had sedentary lifestyle such as long sitting hours showed increased association with hypertension and women who showed endurance and muscle strengthening exercise adherence were associated with decreased odds ratios of hypertension. Particularly, they showed increased association with hypertension along with increment of sitting hours, which is closely related to the level of occupational physical activity.

Previous studies on the effects of occupational physical activity (OPA) on hypertension are inconsistent [19]. OPA has also been shown to lower the risk of hypertension [15]; on the contrary, in the report of increasing risk in black men, it is considered that research on various population groups is necessary in consideration of racial differences, which supports the necessity of this study [20]. In addition, all-cause mortality has been reported as well as hypertension [21]. Potential hypothesis raised to date includes the risk of anaerobic components or injuries [14], adverse social determinants and demanding job characteristics [17], and motion characteristics of OPA which is not helpful for cardiopulmonary function [21].

On the other hand, the mechanism of cardiovascular diseases due to increased sitting hours due to occupation has been explained more clearly. Decreased local contraction of muscles interferes with lipoprotein lipase activity, leading to an increase in triglycerides and a decrease in high-density lipoprotein cholesterol [22]. It is not easy to measure heterogeneous types of OPAs and working environment for the mechanisms by which OPAs pose a risk of hypertension [17], but further research is needed for clarification.

In the aerobic activity adherence group, women doing occupational physical activity of moderate intensity showed increased association with hypertension. Frequency of walking, e.g., days of walking per week, was associated with decreased odds ratios of hypertension in this group.

The relationship between physical activity and hypertension has been reported many times. The previous studies also reported impacts on RCT interventions and risk of coronary heart disease. In the study of Twinamasiko, the mean systolic blood pressure was higher in the sedentary work style group [16]. In a recent meta-analysis of 26 RCTs on physical activities, it was reported that physical activities, even with higher intensity, were associated with reduction in blood pressure in the participants with hypertension [23–26]. Meeting the target of American Physical Activity Guideline resulted in a 14% reduced risk of coronary heart disease compared with inactive people in adults [8]. Also, there was a research identifying the fact that daily physical activity was associated with blood pressure particularly in women [27].

Several studies reported that the mechanisms leading to reducing blood pressure by physical activity were related to cardiac remodelling, endothelial function, decreased oxidative stress, and the inflammation syndrome [28–30]. The mechanism by which aerobic exercise training lowers blood pressure in hypertensive patients has been reported for some time, and the mechanism has been suggested to be related to the sympathetic nerve activity (SNA) [31]. However, evidence suggests that muscle SNA is associated with the central sympathetic nerve activity (SNA) [32]. After 4 months of moderate intensity aerobic exercise training in hypertensive patients with unsuccessful treatment, initial excessive central sympathetic activation decreased, accompanied by decreased muscle SNA and improved arterial baroreflex regulation of SNA [33]. Usual pharmacological approaches for essential hypertension have not focused on regulating central sympathetic outflow, and the emerging therapeutic strategies targeting neurogenic hypertension such as aerobic exercise training, weight loss, stress reduction, central sympatholytics such as moxonidine or rilmenidine, statins, and surgical interventions such as therapeutic renal sympathetic denervation have implications for clinical management of hypertension [32]. Walking was identified to be related to decreased risk of hypertension in participants who showed adherence to aerobic activity, while increased sitting hours could lead to increased risk of hypertension in the participants not adhered to the regular aerobic activity. In Hu's report (2007), hypertensive women had the benefit of reduced cardiovascular mortality from all the type of physical activities such as occupational, leisure-time and daily “walking or cycling to and from work.” Because of these results, walking in women can be consistently recommended [15]. There have been a number of researches reporting that regular or chronic dynamic aerobic and resistance training would be effective on reductions in blood pressure of the patients with hypertension [34–36]. In addition to moderate-vigorous physical activity, the BP lowering effect of reducing sedentary hours through interrupting prolonged sitting was recently reported and “sit less” has been recommended [37]. When this “breaks in prolonged sitting” was performed with morning exercise, it was effective to control BP in obese older adults, particularly in women [38].

Also it was demonstrated that the largest isometric training resulted in reductions of blood pressure in hypertensive patients which was similar to aerobic exercise training [34, 39, 40]. Moreover, the combination of muscle exercises and aerobic activity could be effective in controlling blood pressure [41]. In this study, even the participants without aerobic activities nonadherence demonstrated that regular muscle exercise was associated with decreased risk of hypertension.

There are several limitations in interpreting the results of this study. First, it was not possible to demonstrate any causal relationship between physical activities and hypertension in women aged from 40 to 80 years because of the cross-sectional design of this study. Secondly, there was no significant association between leisure-time activities and hypertension in neither adherence nor nonadherence group. This may be due to the small number of active women in the nonadherence group. Finally, there was no consideration that it was surgical menopause or natural menopause. Information on the risky components in the work activities such as chemical, mechanical injury, or job stress was insufficient to analyze the association between occupational physical activity and hypertension. A more comprehensive study may be necessary to overcome those limitations.

## 5. Conclusion

In the aerobic activity adherence group, women doing occupational physical activity of moderate intensity showed increased association with hypertension. Further research that identifies the dangerous components in occupational physical activity is warranted. Walking seems to have benefits and be appropriate to be recommended. In the aerobic activity nonadherence group, women who had long sitting hours showed increased association with hypertension, and women who showed endurance exercise adherence were associated with decreased odds ratios of hypertension. Decreasing sitting hours and increasing endurance exercise may be helpful for this group.

## Figures and Tables

**Table 1:** Characteristics of the women participated in this study.

Items		*n*	%
Age (years)	40–49 y	1148	27.1
	50–59 y	1210	28.5
	60–69 y	1042	24.6
	≥70 y	841	19.8

Income level	Level 1	940	22.2
	Level 2	1061	25.0
	Level 3	1091	25.7
	Level 4	1142	26.9

Education level	Elementary	1355	32.0
	Middle	568	13.4
	High	1276	30.1
	University	1036	24.4

Hypertension	No	3007	70.9
	Yes	1234	29.1

Aerobic activity adherence^a^ (per week)	No	2560	60.4
	Yes	1681	39.6
Muscle strengthening exercises adherence^b^ (per week)	Never	3560	83.9
	≤4	465	11.0
	5∼7 days	215	5.1

OPA vigorous intensity (per week)	Never	4219	99.5
	≤4	12	0.3
	5∼7 days	10	0.2

OPA moderate intensity (per week)	Never	4061	95.8
	≤4	108	2.5
	5∼7 days	72	1.7

Physical activity days with moving location (per week)	Never	1801	42.5
	≤4	1101	26.0
	5∼7 days	1339	31.6

LPA vigorous intensity (per week)	Never	4011	94.6
	≤4	162	3.8
	5∼7 days	68	1.6

LPA moderate intensity (per week)	Never	3436	81.0
	≤4	577	13.6
	5∼7 days	228	5.4

Sitting hours (per day)	≤6 hours	1831	43.2
	≤12 hours	2028	47.8
	≥13 hours	382	9.0

Walking (days per week)	Never	823	19.4
	≤4 days	1578	37.2
	≥5 days	1838	43.3

Sleeping hours in weekdays (per week)	<7 hours	2594	61.2
	≥7 hours	1647	38.8

Sleeping hours in weekends (per week)	<7 hours	1989	46.9
	≥7 hours	2252	53.1

Alcohol consumption (within 1 year)	No experience	884	20.8
	No drink within recent 1 year	834	19.7
	≤4 times (months)	2147	50.6
	≥5 times (months)	376	8.9

Smoking status	Never	3933	92.7
	Former smoker	162	3.8
	Current smoker	145	3.4

^a^Aerobic activity adherence: 2.5-hour moderate intensity physical activity per week or 1.25-hour vigorous intensity physical activity per week. ^b^Muscle strengthening exercises adherence: push-up, sit-up, dumbbell, or other types of muscle exercises more than 2 days during 1 week recently. OPA: occupational physical activity; LPA: leisure-time physical activity.

**Table 2:** General characteristics and physical activity status due to aerobic activity adherence.

Items		Total	Nonaerobic (*n* = 2,560)		Aerobic (*n* = 1,681)		*p* value
		*n*	*n*	%	*n*	%	
Age (years)	40–49 y	1148	624	24.4	524	31.2	<0.001^*∗∗*^
	50–59 y	1210	660	25.8	550	32.7	
	60–69 y	1042	640	25.0	402	23.9	
	≥70 y	841	636	24.8	205	12.2	

Income level	Level 1	940	611	23.9	329	19.6	<0.001^*∗∗*^
	Level 2	1061	660	25.8	401	23.9	
	Level 3	1091	640	25.1	451	26.8	
	Level 4	1142	643	25.2	499	29.7	

Education level	Elementary	1355	970	38.0	385	22.9	<0.001^*∗∗*^
	Middle	568	351	13.7	217	12.9	
	High	1276	703	27.5	573	34.1	
	University	1036	531	20.8	505	30.1	

Hypertension	No	3007	1689	66.0	1318	78.4	<0.001^*∗∗*^
	Yes	1234	871	34.0	363	21.6	

Aerobic activity adherence^a^ (per week)	No	3560	2265	88.5	1295	77.0	<0.001^*∗∗*^
	Yes	465	199	7.8	266	15.8	
	Never	215	95	3.7	120	7.1	

OPA vigorous intensity (per week)	≤4 days	4219	2557	99.9	1662	98.9	<0.001^*∗∗*^
	5∼7 days	12	2	0.1	10	0.6	
	Never	10	1	0.0	9	0.5	

OPA moderate intensity (per week)	≤4 days	4061	2533	98.9	1528	90.9	<0.001^*∗∗*^
	5∼7 days	108	25	1.0	83	4.9	
	Never	72	2	0.1	70	4.2	

Physical activity days with moving location (per week)	≤4 days	1801	1592	62.2	209	12.4	<0.001^*∗∗*^
	5∼7 days	1101	710	27.7	391	23.3	
	Never	1339	258	10.1	1081	64.3	

LPA vigorous intensity (per week)	≤4 days	4011	2550	99.6	1461	86.9	<0.001^*∗∗*^
	5∼7 days	162	10	0.4	152	9.0	
	Never	68	0	0.0	68	4.0	

LPA moderate intensity (per week)	≤4 days	3436	2412	94.2	1024	60.9	<0.001^*∗∗*^
	5∼7 days	577	138	5.4	439	26.1	
	Never	228	10	0.4	218	13.0	

Sitting hours (per day)	≤6 hours	1831	1003	39.2	828	49.3	<0.001^*∗∗*^
	≤12 hours	2028	1290	50.4	738	43.9	
	≥13 hours	382	267	10.4	115	6.8	

Walking (days per week)	Never	823	731	28.6	92	5.5	<0.001^*∗∗*^
	≤4 days	1578	1126	44.0	452	26.9	
	5∼7 days	1838	702	27.4	1136	67.6	

Sleeping hours in weekdays (per week)	<7 hours	2594	1505	58.8	1089	64.8	<0.001^*∗∗*^
	≥7 hours	1647	1055	41.2	592	35.2	

Sleeping hours in weekends (per week)	<7 hours	1989	1189	46.4	800	47.6	0.465
	≥7 hours	2252	1371	53.6	881	52.4	

Alcohol consumption (within 1 year)	No experience	884	584	22.8	300	17.8	<0.001^*∗∗*^
	No drink within recent 1 year	834	535	20.9	299	17.8	
	≤4 times (months)	2147	1210	47.3	937	55.7	
	≥5 times (months)	376	231	9.0	145	8.6	

Smoking status	Never	3933	2357	92.1	1576	93.8	0.091
	Former smoker	162	103	4.0	59	3.5	
	Current smoker	145	99	3.9	46	2.7	

^a^Aerobic activity adherence: 2.5-hour moderate intensity physical activity per week or 1.25-hour vigorous intensity physical activity per week. ^b^Muscle strengthening exercises adherence: push-up, sit-up, dumbbell, or other types of muscle exercises more than 2 days during 1 week recently. OPA: occupational physical activity; LPA: leisure-time physical activity. ^*∗*^*p* < 0.05; ^*∗∗*^*p* < 0.01.

**Table 3:** Comparison of clinical characteristics according to the aerobic activity adherence.

	Aerobic activity nonadherence group (*n* = 2,560)		Aerobic activity adherence group (*n* = 1,681)		*p* value
	Mean	SD	Mean	SD	
Mean SBP	122.05	17.73	119.25	17.42	<0.001^*∗∗*^
Mean DBP	74.58	9.51	75.17	9.32	0.048^*∗*^
BMI	24.12	3.57	23.79	3.34	0.002^*∗*^
Total cholesterol	198.51	39.27	199.82	38.15	0.294
HDL cholesterol	52.48	12.53	54.61	12.66	<0.001^*∗∗*^
Triglyceride	132.38	100.99	117.13	77.88	<0.001^*∗∗*^
AST (GOT)	22.43	9.65	21.73	8.54	0.016^*∗*^
ALT (GPT)	19.85	12.98	18.84	12.08	0.011^*∗*^

SD: standard deviation; SBP: systolic blood pressure; DBP: diastolic blood pressure; BMI: body mass index; GOT: glutamate oxaloacetate transaminase; GPT: glutamate-pyruvate transaminase. ^*∗*^*p* < 0.05; ^*∗∗*^*p* < 0.01.

**Table 4:** Association between physical activity and hypertension in the aerobic activity nonadherence group.

Items		Unadjusted OR	*p* value	Adjusted^a^ OR	*p* value
Muscle strengthening exercises adherence^b^ (per week)	Never		<0.001^*∗∗*^		0.034^*∗*^
	≤4	0.468	<0.001^*∗∗*^	0.554	0.010^*∗*^
	5∼7 days	0.830	0.433	0.850	0.570

OPA vigorous intensity (per week)	Never		1.000		1.000
	≤4		0.999		0.999
	5∼7 days	0.000	1.000	0.000	1.000

OPA moderate intensity (per week)	Never		0.247		0.550
	≤4	0.446	0.116	0.533	0.282
	5∼7 days	2.797	0.570	1.577	0.847

Physical activity days with moving location (per week)	Never		0.131		0.173
	≤4	1.173	0.134	1.000	0.999
	5∼7 days	1.301	0.091	1.410	0.069

LPA vigorous intensity (per week)	Never				
	≤4	0.262	0.212	0.158	0.149
	5∼7 days				

LPA moderate intensity (per week)	Never		0.493		0.441
	≤4	0.782	0.254	1.318	0.281
	5∼7 days	1.247	0.757	1.821	0.477

Sitting hours (per day)	≤6 hours		<0.001^*∗∗*^		0.005^*∗*^
	7∼12 hours	1.211	0.041^*∗*^	1.161	0.190
	≥13 hours	2.275	<0.001^*∗∗*^	1.849	0.001^*∗*^

Walking (days per week)	Never		0.096		0.203
	≤4	0.802	0.048^*∗*^	1.187	0.213
	5∼7 days	0.953	0.704	1.310	0.078

Sleeping hours in weekdays (per week)	≥7 hours	2.007	<0.001^*∗∗*^	1.329	0.065

Sleeping hours in weekends (per week)	≥7 hours	0.591	<0.001^*∗∗*^	0.942	0.698

Alcohol consumption (within 1 year)	No experience		<0.001^*∗∗*^		0.776
	No drink within recent 1 year	0.680	0.002^*∗*^	0.895	0.474
	≤4 times (months)	0.491	<0.001^*∗∗*^	0.912	0.497
	≥5 times (months)	0.475	<0.001^*∗∗*^	0.807	0.337

Smoking status	Never		0.262		0.246
	Former smoker	0.846	0.465	0.776	0.372
	Current smoker	0.690	0.134	0.637	0.144

^a^Adjusted by covariates (controlling age, income level, education levels, smoking status, and alcohol consumption) in multiple logistic regression analysis. ^b^Muscle strengthening exercises adherence: push-up, sit-up, dumbbell, or other types of muscle exercises more than 2 days during 1 week recently. OR: odds ratio; OPA: occupational physical activity; LPA: leisure-time physical activity. ^*∗*^*p* < 0.05; ^*∗∗*^*p* < 0.01.

**Table 5:** Association between physical activity and hypertension in the aerobic activity adherence group.

Items		Unadjusted OR	*p* value	Adjusted^a^ OR	*p* value
Muscle strengthening exercises adherence^b^ (per week)	Never		0.879		0.527
	≤4	0.928	0.693	1.292	0.259
	5∼7 days	1.069	0.789	1.067	0.819

OPA vigorous intensity (per week)	Never		0.541		0.698
	≤4	0.890	0.891	0.558	0.572
	5∼7 days	0.300	0.270	0.422	0.516

OPA moderate intensity (per week)	Never		0.050^*∗*^		0.123
	≤4	1.515	0.123	1.931	0.048^*∗*^
	5∼7 days	0.509	0.074	0.859	0.725

Physical activity days with moving location (per week)	Never		0.623		0.861
	≤4	1.203	0.467	1.073	0.813
	5∼7 days	1.269	0.331	1.153	0.619

LPA vigorous intensity (per week)	Never		0.007^*∗*^		0.474
	≤4	0.537	0.030^*∗*^	0.852	0.627
	5∼7 days	0.314	0.017^*∗*^	0.538	0.251

LPA moderate intensity (per week)	Never		0.101		0.338
	≤4	0.701	0.032^*∗*^	0.798	0.256
	5∼7 days	0.906	0.652	1.163	0.552

Sitting hours (per day)	≤6 hours		0.323		0.845
	7∼12 hours	1.211	0.133	1.002	0.991
	≥13 hours	1.102	0.692	0.841	0.576

Walking (days per week)	Never		0.002^*∗*^		0.103
	≤4	0.410	0.001^*∗*^	0.532	0.049^*∗*^
	5∼7 days	0.436	0.001^*∗*^	0.530	0.037^*∗*^

Sleeping hours in weekdays (per week)	≥7 hours	1.335	0.076	1.182	0.409

Sleeping hours in weekends (per week)	≥7 hours	0.656	0.007^*∗*^	0.844	0.380

Alcohol consumption (within 1 year)	No experience		<0.001^*∗∗*^		0.859
	No drink within recent 1 year	0.756	0.135	1.134	0.580
	≤4 times (months)	0.545	<0.001^*∗∗*^	1.049	0.806
	≥5 times (months)	0.403	0.001^*∗*^	0.863	0.662

Smoking status	Never		0.394		0.264
	Former smoker	1.538	0.172	1.878	0.109
	Current smoker	1.020	0.961	0.890	0.798

^a^Adjusted by covariates (controlling age, income level, education levels, smoking status, and alcohol consumption) in multiple logistic regression analysis. ^b^Muscle strengthening exercises adherence: push-up, sit-up, dumbbell, or other types of muscle exercises more than 2 days during 1 week recently. OR: odds ratio; OPA: occupational physical activity; LPA: leisure-time physical activity. ^*∗*^*p* < 0.05; ^*∗∗*^*p* < 0.01.

## Data Availability

The data used to support the findings of this study are available from the website of the Korea National Health and Nutrition Examination Survey (https://knhanes.cdc.go.kr/knhanes/main.do).
